# Using extracorporeal membrane oxygenation to rescue acute myocardial infarction with cardiopulmonary collapse: the impact of early coronary revascularization

**DOI:** 10.1186/1749-8090-8-S1-O82

**Published:** 2013-09-11

**Authors:** MY Wu

**Affiliations:** 1 Department of Cardiac Surgery, Chang Gung Memorial Hospital, Taoyuan, Taiwan

## Background

To investigate the therapeutic impact of combining extracorporeal membrane oxygenation (ECMO) and early coronary revascularization on acute myocardial infarction (AMI)-induced cardiopulmonary collapse.

## Methods

This retrospective study included 35 consecutive patients rescued by ECMO for AMI -induced cardiopulmonary collapse in a single institution between June 2003 and December 2011. Coronary revascularization was performed soon after ECMO initiation. Percutaneous coronary intervention (PCI) was the primary revascularization strategy. Coronary artery bypass grafting (CABG) was performed if an unsuitable anatomy or unsatisfactory result of PCI. Comparisons were performed in groups with different revascularization strategies and outcomes.

## Results

Among the 35 patients, 16 underwent CABG and 1 was bridged to transplant after CABG. Compared to patients receiving PCI only, the CABG group showed similar results in ECMO weaning (58% vs. 69%, p=0.51), hospital discharge (32% vs. 50%, p=0.27), and left ventricular ejection fraction before discharge (45% vs. 49%, p=0.92). Regardless of revascularization strategies, this protocol achieved an ECMO-weaning rate of 63% and a hospital discharge rate of 40%. Dialysis-dependent acute renal failure (OR 5.4, 95% CI 1.1–27.5) and profound anoxic encephalopathy (OR 5.4, 95% CI 1.1–27.5) predicted non-weaning of ECMO. Age > 60 years (OR 7.3, 95% CI 1.1–51.0) and profound anoxic encephalopathy (OR 24.6, 95% CI 2.3–263.0) predicted in-hospital mortality. The major cardiovascular adverse effect (MACE) –free survival was 77% in the first year after discharge.

## Conclusions

Early revascularization on ECMO is practical to preserve myocardial viability and bridge patients collapsing with AMI to recovery.

